# Intracellular Macromolecular Crowding within Individual Stress Fibers Analyzed by Fluorescence Correlation Spectroscopy

**DOI:** 10.1007/s12195-024-00803-4

**Published:** 2024-06-07

**Authors:** Aria Buenaventura, Takumi Saito, Taiga Kanao, Daiki Matsunaga, Tsubasa S. Matsui, Shinji Deguchi

**Affiliations:** 1 Division of Bioengineering, Osaka University, Toyonaka, 560-0043 Japan; 2 Graduate School of Biomedical Engineering, Tohoku University, Sendai, 980-0812 Japan; 3 Department of Molecular Biophysics and Biochemistry, Yale University, New Haven, USA; 4 Nanobiology Institute, Yale University, West Haven, USA

**Keywords:** FCS, Protein diffusion, Macromolecular crowding, Nucleus, Stress fibers, Autocorrelation

## Abstract

**Introduction:**

The diffusion of cell components such as proteins is crucial to the function of all living cells. The abundance of macromolecules in cells is likely to cause a state of macromolecular crowding, but its effects on the extent of diffusion remain poorly understood.

**Methods:**

Here we investigate the diffusion rate in three distinct locations in mesenchymal cell types, namely the open cytoplasm, the stress fibers in the open cytoplasm, and those below the nucleus using three kinds of biologically inert green fluorescent proteins (GFPs), namely a monomer, dimer, and trimer GFP. Fluorescence correlation spectroscopy (FCS) was used to determine the diffusion coefficients.

**Results:**

We show that diffusion tends to be lowered on average in stress fibers and is significantly lower in those located below the nucleus. Our data suggest that the diffusive properties of GFPs, and potentially other molecules as well, are hindered by macromolecular crowding. However, although the size dependence on protein diffusion was also studied for monomer, dimer, and trimer GFPs, there was no significant difference in the diffusion rates among the GFPs of these sizes. These results could be attributed to the lack of significant change in protein size among the selected GFP multimers.

**Conclusion:**

The data presented here would provide a basis for better understanding of the complex protein diffusion in the nonuniform cytoplasm, shedding light on cellular responses to mechanical stress, their local mechanical properties, and reduced turnover in senescent cells.

## Introduction

One approach to understanding the inner workings of living cells and their resulting biophysical properties is through the analysis of intracellular diffusion, i.e., how molecules are transported within the cells. Although typically represented by Brownian motion, several elements alter the normal diffusion of proteins, preventing the full comprehension of their motion [1–3]. To explain this anomalous behavior, many studies describe a phenomenon known as macromolecular crowding that affects diffusion with high concentrations of macromolecules occupying the intracellular space [4, 5]. Recently, studies on the effects of macromolecular crowding on biological thermodynamics and kinetics have contributed to many fields including biophysics, drug delivery, and soft matter physics [6–8]. However, due to the unpredictability of diffusive patterns due to this crowding, effects on molecular processes are far from being fully understood, and more experimental data are necessary to supplement theoretical causes for the anomalous diffusion [9–12].

**Figure 1:**
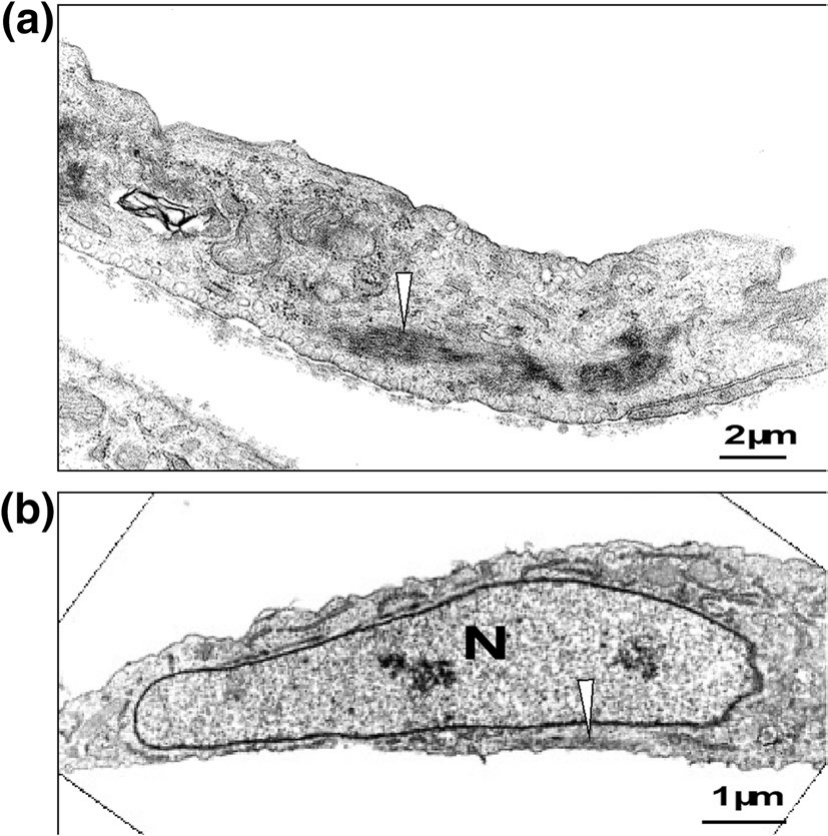
Transmission electron microscopy of electron dense region in bovine aortic smooth cells. **a** and **b** Two examples of stress fibers (arrows) are shown, in which they are located in the cytoplasm away from the nucleus (**a**) or below the nucleus (**b**). N denotes the nucleus

According to studies in vitro and in vivo, the main cause of anomalous diffusion is crowding not only caused by other moving proteins and nucleic acids but also by fixed obstacles such as the actin cytoskeleton [13, 14]. Among such cytoskeletal structures, it is of importance to study the crowding effects of stress fibers, which are thick actin bundles known to be essential for cellular adaptation to extracellular cues to maintain intracellular homeostasis [15–18]. To enable adaptive responses, stress fibers promptly assemble and disassemble in response to stimuli, but it remains unclear how such thick stress fibers interact with surrounding diffusive signaling molecules. Although individual stress fibers have a variable width of approximately 1 μm, with the constituent individual actin filaments at approximately 5.5 nm in width, they appear as electron dense regions in electron microscopy reflecting condensed structures because their components include myosin II and α-actinin (Fig. 1) [19–22]. Based on these considerations, we hypothesized that molecules in regions with densely packed stress fibers might behave with a lower diffusion rate because of the effect of macromolecular crowding at the subcellular scale. In senescent cells, thickened stress fibers are highly expressed to lead to reduced turnover and energy consumption [23], which are also consistent with the hypothesis that diffusion in the crowded cell interior is limited due to potential macromolecular crowding. In fact, it is known that the density of proteins is decreased within senescent cells even at the scale of individual cell organelles [24, 25], which is accompanied with lowered turnover with unknown mechanisms. To test the hypothesis, here we investigate the diffusion within individual stress fibers using biologically inert green fluorescent proteins (GFPs), which have typically been used for the analysis of diffusion [1, 9, 11, 26, 27].

To quantitatively evaluate the diffusion of molecules, a method called fluorescence correlation spectroscopy (FCS) is often used [4, 28–31]. By analyzing the fluctuations in fluorescence intensity that labels the molecules in a small volume defined by a focused laser spot, the diffusion coefficient of these molecules is extracted. The diffusion coefficient is obtained from an autocorrelation function, which describes the relationship between the individual particle motions and the resulting diffusion. The FCS method is useful in many fields aside from the measurement of diffusion, e.g., in quantification of aggregation, receptor-ligand interactions, and DNA hybridization [4, 32]. Because of the small volume used in FCS measurement, the diffusion of small fluorescent molecules in structures contributing to macromolecular crowding could be thoroughly observed [28–30].

**Figure 2:**
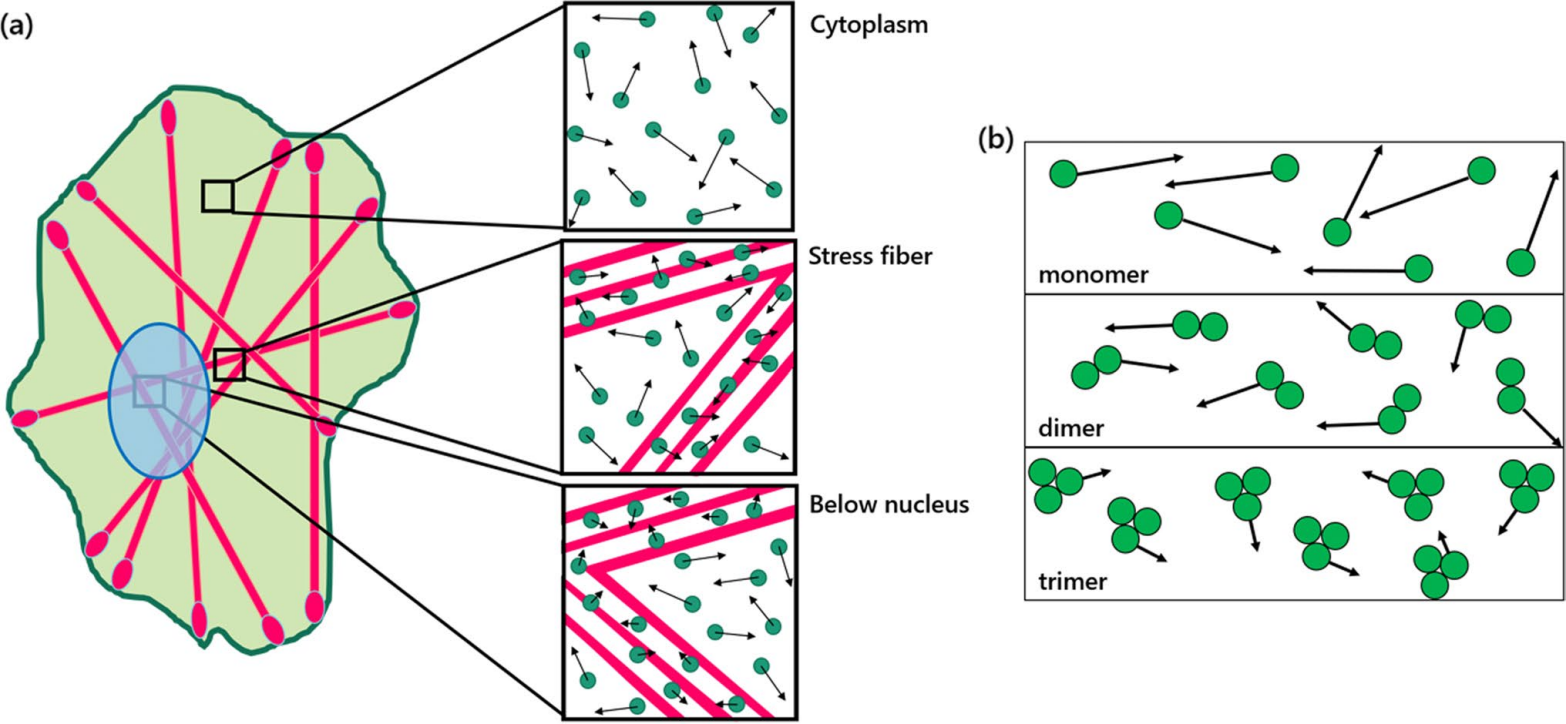
Scheme of the research design. Diffusion of green fluorescent proteins (GFPs) in the cell as affected by macromolecular crowding of the stress fibers. **a** Cells are supposed to be transfected with mClover2, mRuby-Lifeact, and stained with Hoechst. GFP molecules move in three distinct locations, namely, in the cytoplasm, in the stress fibers, and in the stress fibers below the nucleus. When located in stress fibers, GFP movement is limited, thus lowering their rate of diffusion. Similarly, stress fibers and the actin meshwork restrict the movement of GFP below the nucleus. **b** Three different GFP types, namely, the monomer, dimer, and trimer GFPs. It is predicted that larger GFP molecules will diffuse slower than smaller ones

**Figure 3:**
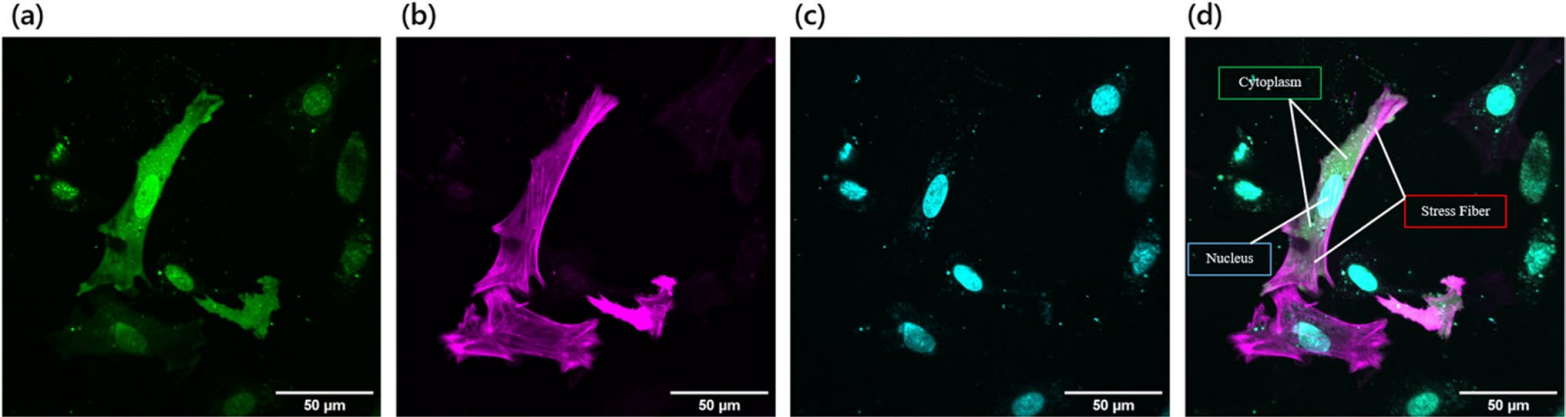
Confocal images of representative cells to analyze the intracellular colocalization. **a** GFP (mClover2, a monomer). **b** Stress fibers (mRuby Lifeact). **c** The nucleus (Hoechst staining). **d** Merged image. Scale, 50 μm

The areas we focused in this study were the open cytoplasm, the stress fibers in the open cytoplasm, and those below the nucleus (Figs. 2 and 3). First, because the cytoplasm acts as a relatively free space in which GFP diffusion most resembles Brownian diffusion, diffusion in this area may be used to compare how quickly or slowly GFP diffuses in other areas. Secondly, the behavior of molecules inside stress fibers is of interest given that we aim to observe crowding effects of immobile structures. Lastly, diffusion behavior in the area below the nucleus is to be considered, as there is a supposed spatial confinement imposed by the nucleus onto this area, possibly causing a more compact actin meshwork, subsequently enhancing macromolecular crowding and reducing the molecular diffusion rate [33–35].

Furthermore, we also take into consideration the influence of varying the molecular mass or size on diffusion. Theoretical and experimental studies thus far usually only focus on the diffusive behavior of spherical molecules, even if the cell is made up of molecules with more complex shapes and sizes [36]. Macromolecular crowding may have different effects on particles with varying shapes and sizes due to their susceptibility to being trapped by the surrounding cytoskeletal structure [36, 37]. We thus performed experiments using three types of GFP, namely a monomer, dimer, and trimer GFP, but it must be noted that the effects of the linkers used on the overall dimer and trimer shape and size were noted to be negligible.

## Materials and Methods

### Cell Culture

Rat aortic smooth muscle cell lines (A7r5, ATCC) were cultured with low-glucose (1.0 g/L) Dulbecco’s Modified Eagle Medium (Wako) containing 10% (v/v) heat-inactivated fetal bovine serum (SAFC Biosciences) and 1% penicillin-streptomycin (Wako) in a 5% CO_2_ incubator at 37 °C.

### Plasmid Construction and Transfection

The plasmids of 2-mClover2 and 3-mClover2 were constructed by Gibson assembly of two and three fragments obtained by amplification of the plasmid mClover2-C1 (Addgene plasmid #54577, a gift from Michael Davidson) with the following primers (Supporting Material; [38]):

mClo_BackBone_tail_Fwd: 5’-TCCGGACTCAGATCTCGAG-3’

mClo_BackBone_tail_Rev: 5’-CTTGTACAGCTCGTCCATG-3’

2_mClo_Fwd: 5’-GCATGGACGAGCTGTACAAGAGAAGCATGGTGAGCAAG-3’

2_mClo_Rev: 5’-TGCTCACCATGCTTCTCTTGTACAGCTC-3’

3_mClo_Fwd: 5’-CAAGAGAAGCATGGTGAGCAAGGGCGAG-3’

3_mClo_Rev: 5’-GCTCGAGATCTGAGTCCGGACTTGTACAGCTCGTCCATGC-3’.

The plasmids of mRuby2-Lifeact [39] and of either 2-mClover2-C1, 2-mClover2, or 3-mClover2 were transfected to cells 8 hours after the seeding of cells using Lipofectamine LTX and plus Reagent (Thermo Fischer Scientific) according to the manufacturer’s instructions. The Hoechst 33342 (ThermoFischer Scientific) stain was used 15 minutes prior to the experiment.

### Fluorescence Correlation Spectroscopy

The fluctuations of the fluorescence intensity are analyzed as a function of time $I(t)$ based on FCS [29]. The fluctuations are dependent on the particle’s mass and concentration, allowing the fluorescence signals to be transformed into diffusion values [40]. To statistically analyze the spontaneous fluctuations of fluorescence, the time autocorrelation function is computed. This function provides information on the different diffusion properties. Although the detection volume size in the FCS method is limited by diffraction, the autocorrelation function can be altered by processes on small spatial scales [31]. For FCS experiments, cells were cultured on a glass-bottom dish (φ 27 mm, No. 1S, #3970-035, Iwaki and transfected with the plasmids for 24 hours. The intensity profiles of the emitted fluorescence of the GFP were obtained by using the FV1000 confocal laser scanning microscope (Olympus with a 60X oil immersion objective lens (NA = 1.42 at the outside or inside of the cells cultured on a glass-bottom dish. The measurement was performed using live A7r5 cells on a stage incubator (Tokai Hit,37 °C, 5% CO_2_).

The autocorrelation function $G$ of the fluorescence intensity $I(t)$ is described as a function of the lag time $\tau$ as

1 $$G\left(\tau \right)=\frac{\langle \delta I(t)\delta I(t+\tau )\rangle }{{\langle I\rangle }^{2}}$$

where $\delta I(t)$ (= $I\left(t\right)-\langle I\rangle$) represents the fluctuation of the intensity detected every 2 nanoseconds. The autocorrelation function can be expressed in terms of the diffusion coefficient $D$ and the average particle number $\overline{N }$ as follows

2 $$G\left(\tau \right)=\frac{1}{\overline{N} }{\left(1+\frac{4D\tau }{{{W}_{xy}}^{2}}\right)}^{-1}{\left(1+\frac{4D\tau }{{{W}_{z}}^{2}}\right)}^{-1}$$

where ${W}_{xy}$ and ${W}_{z}$ represent the transverse and axial confocal volume parameters, respectively, determined from the numerical aperture of 1.42, wavelength of 488 nm, and pinhole size of 110 μ m of the current setup [41]. $D$ and $\overline{N }$ are then determined from the measured data of the $G$–$\tau$ relationship by the least square method.

To filter the noise out of the data, we used the simple moving average method on the data resulting from the autocorrelation function. This method eliminates short-term fluctuations and improves the signal-to-noise ratio of the data by taking the average of a fixed number of items in a time series, which move through the series by dropping the top items of the previous averaged group and adding the next in each successive average [41–46].

3 $${x}_{i}=\frac{1}{N}\sum_{i=1}^{N}{x}_{i}$$

This method reduces the amount of variation in the data as well as the effect of extreme values.

### Transmission Electron Microscopy

To observe the microstructure of cells, transmission electron microscopy was performed. Bovine aortic smooth muscle cells [19] were fixed on ice with 2.5% glutaraldehyde, 2% paraformaldehyde, and 0.5% tannic acid in 0.1 M sodium cacodylate buffer for 24 hours. The fixed samples were rinsed with 0.1 M sodium cacodylate buffer for 1 hour, scraped off from the dish, post-fixed at 4 °C with 1% osmium tetroxide in the same buffer for 2 hours, dehydrated using an ethanol series (60%, 70%, 80%, 90% and 100%), infiltrated with n-butyl glycidyl ether, embedded in Epon812, and cut with ultramicrotomes. Ultra-thin sections were mounted onto copper grids, stained with aqueous uranyl acetate and lead citrate, and viewed using a transmission electron microscope (H-7100, Hitachi) at an accelerating voltage of 75 kV.

### Statistical Analysis

Unless otherwise stated, data are expressed as the mean ± standard deviation of *n* = 41 cells from *N* = 7 independent experiments. A normality test was used to check whether an unpaired Student’s *t*-test could be used. In the intensity dataset used, normality was not rejected so that the Student’s *t*-test was used, with a significance level of *p* < 0.01 (**). On the other hand, in the diffusion coefficient dataset, normality was rejected for the cytoplasm and stress fiber datasets. Differences were calculated based on the Mann-Whitney nonparametric test, with a significance level of *p* < 0.05 (*) and p < 0.01 (**). The outliers were omitted only in the statistical analyses, but were included in the plots shown.

## Results

**Figure 4:**
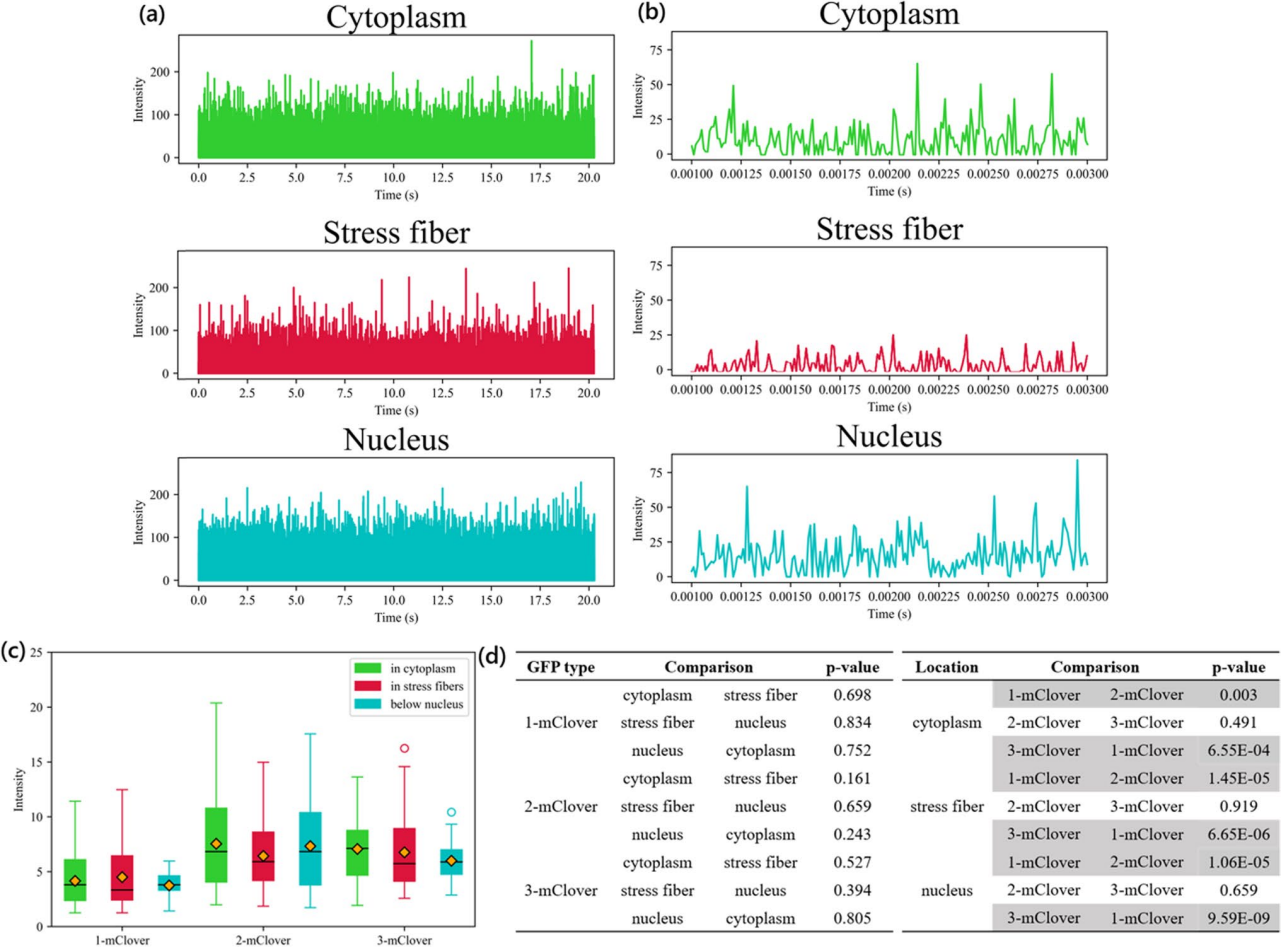
Fluctuations in GFP intensity signal. **a** Average fluctuations of fluorescence intensity of GFP measured in the open cytoplasm, stress fibers in the open cytoplasm, and in stress fibers below the nucleus over 20 s. **b** A closer look on the average fluctuations of fluorescence intensity over 2 ms. **c** Intensity values for GFP in the three locations. **d** Table of the statistical analysis performed, where the values in gray highlight the comparisons between datasets with a significance level of *p* < 0.01

For this study, *N* = 7 sets of independent experiments were conducted, in which a total of *n* = 41 different cells were analyzed. We selected cells that apparently express the same level of GFP by initially observing the intensity before FCS measurement. For each cell, point scanning was done, where *n* = 3 to 6 intensity data points were taken from each area, i.e., in the open cytoplasm (Cytoplasm), in the stress fibers in the cytoplasm (Stress fibers), and in the stress fibers below the nucleus (Nucleus) (Fig. 4). Thus, in total, around 160 different points were analyzed for each location group. The A7r5 cells selected for measurement were approximately 100 μm in size. To minimize the effect of the inhomogeneity of the cell interior, the measurements for the open cytoplasm were taken at a distance of at least 10 μm away from the nucleus. The time interval of each measurement of fluorescence intensity for the FCS experiment was 2 μs, and the total time elapsed for the whole of each experiment was 20 seconds. Photon counting as opposed to analog counting was done on the cells, with the laser power of the 488 nm laser set at 2% and the pinhole size set at 110 μm. Fig. 4a shows typical intensity data of one point scan for 20 seconds, while Fig. 4b shows a shorter scale of 3 μs to clearly show the variations in intensity.

Fig. 4c shows a comparison of the intensity profiles and the relationship between the fluorescence intensity and the subcellular region where the GFP is located. Measurements were done for the monomer, dimer, and trimer GFPs in the three subcellular regions of study. General observations show that monomer GFPs emitted a lower intensity than dimer and trimer GFPs. Statistical analysis was performed, and the resulting *p*-values obtained are listed in Fig. 4d. Here, the gray rows highlight the *p*-values lower than 0.01. There were no significant statistical differences observed when comparing the datasets for the three cellular locations (Cytoplasm, Stress fibers, and Nucleus) or when comparing the dimer and trimer datasets. However, a comparison of the monomer with either the dimer or trimer GFP datasets resulted in a *p*-value lower than 0.01.

**Figure 5:**
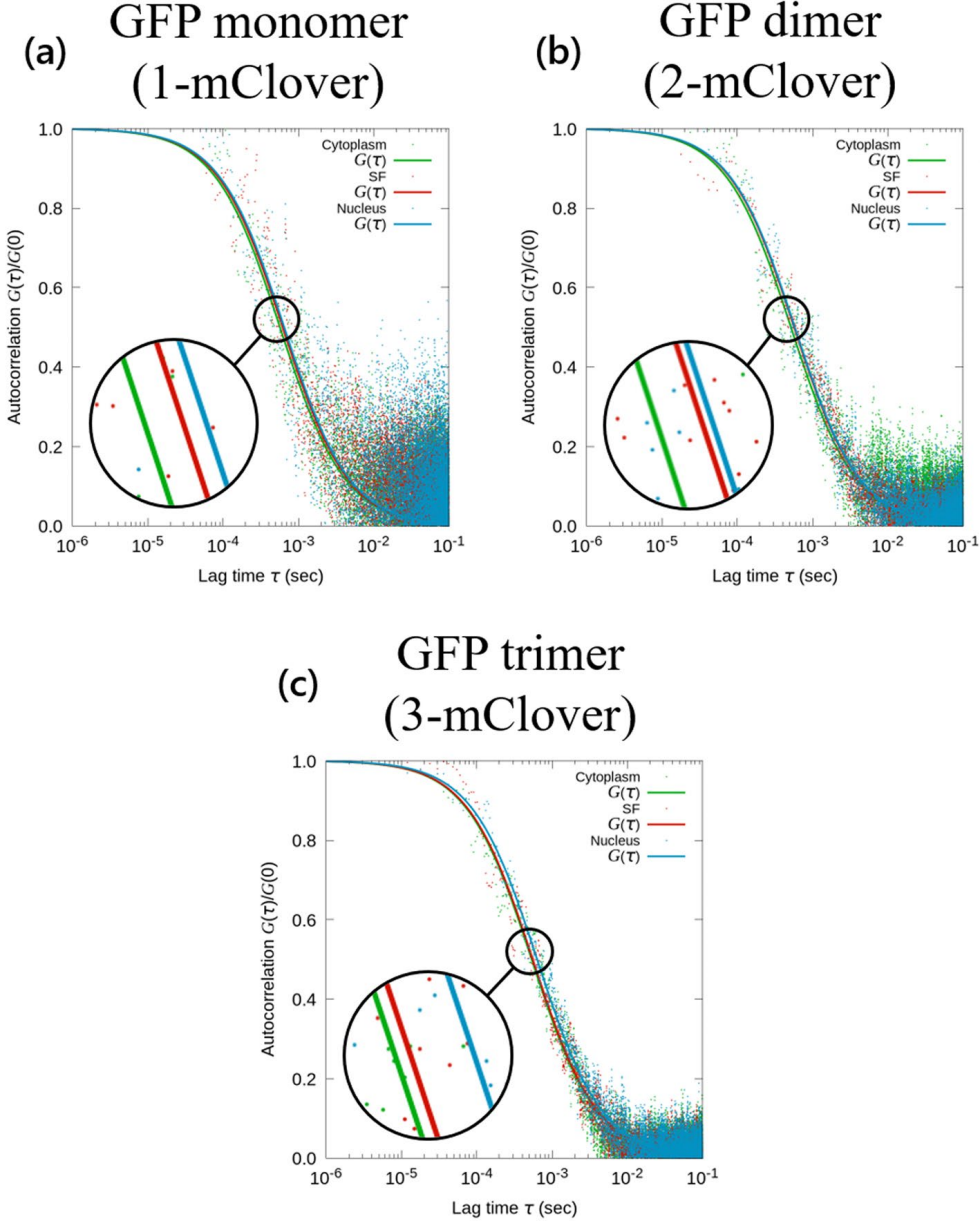
FCS analysis. Fluorescence autocorrelation function of three GFP types, namely the **a** GFP monomer, **b** GFP dimer, and (c) GFP trimer. Each autocorrelation graph shows three fitted curves representing the average trend for the respective GFP in the open cytoplasm (green), in the stress fibers (red), and below the nucleus (blue)

The autocorrelation function in Eq. (1) gives more meaning to the raw intensity data in Fig. 4, in which the autocorrelation is scatter-plotted as a function of the lag time in Fig. 5. The resulting data was then fitted for the lag time range of the decay component from 5 × 10^−5^ to 5 × 10^−3^ seconds using Eq. (2) to determine the diffusion coefficient. For this analysis, the transverse and axial confocal volume parameters *W*_*xy*_ and *W*_*z*_ used were 0.174 μm and 2.82 μm, respectively. Looking at the autocorrelation decay function for the three cellular areas of study, its characteristic decay time (translational diffusion time) was τ_*trans*_ = 4.95 μs. Translational diffusion time corresponds to the average dwell time of the target molecules in the measurement volume. Longer translational diffusion times would therefore indicate slower translational diffusion. In Fig. 5, for the autocorrelation functions for all three GFP types, the decay time was the fastest for the GFP in the cytoplasm and the slowest for the GFP below the nucleus.

**Figure 6:**
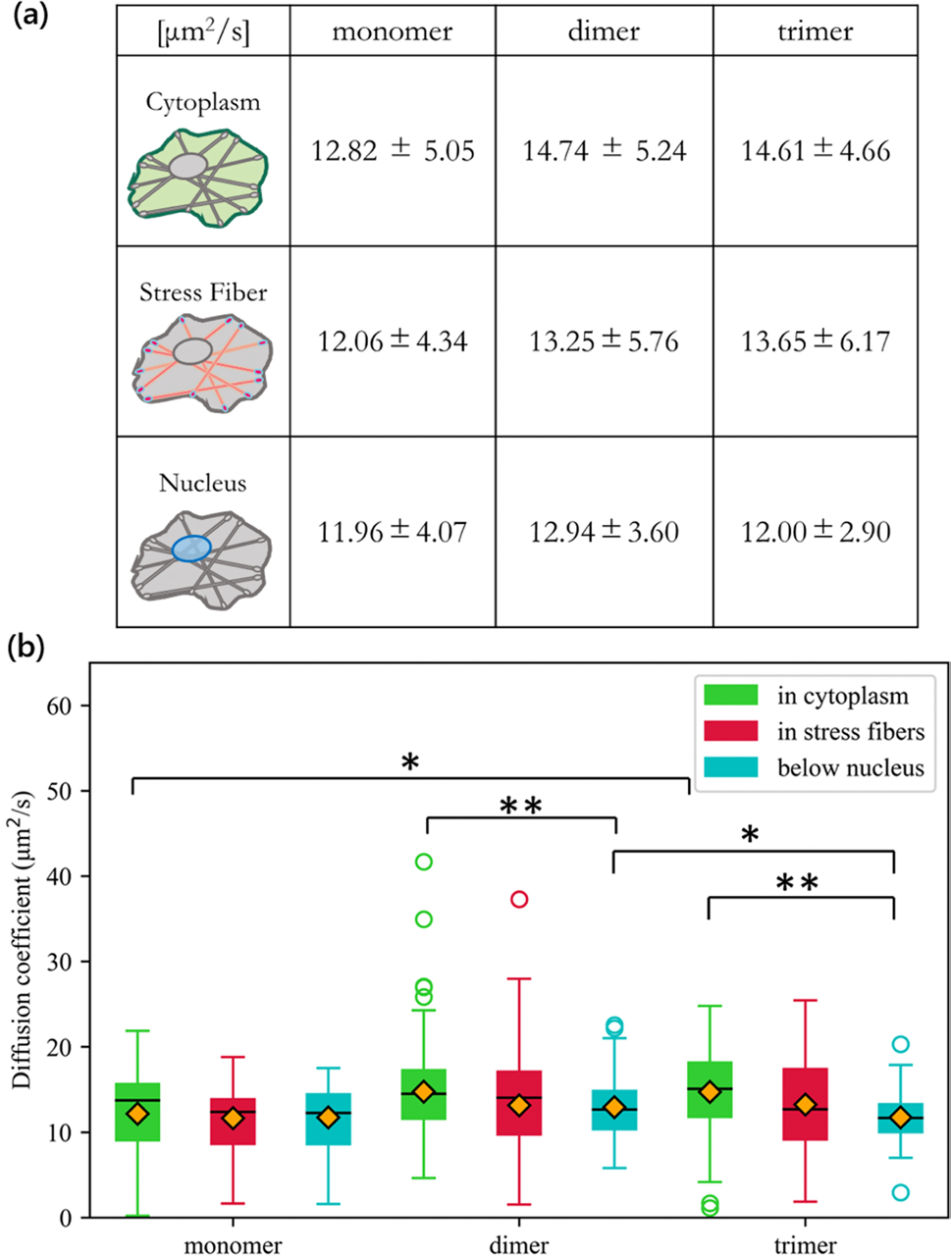
Macromolecular crowding effect on diffusion coefficient. **a** A complete comparison of the average diffusion coefficients across the three locations in the cell, namely the open cytoplasm, the stress fibers, and the area below the nucleus, and across the three different GFP types, namely the monomer, dimer, and trimer GFP. **b** Box-plot comparison of the average diffusion coefficients for the three GFP types

In Fig. 6a, diffusion was observed in the three areas: the open cytoplasm, stress fibers, and those below the nucleus to observe the effect of crowdedness on diffusion. Further, the diffusion of the three GFP types were compared in Fig. 6b. The results show that for all three GFP types, the highest diffusion rate was observed for the GFP in the open cytoplasm (green). This was followed by the GFP diffusing in the stress fibers (red), and then by the GFP diffusing in the stress fibers below the nucleus (blue).

Statistical analysis showed that, while the average diffusion coefficient of the GFPs diffusing in the cytoplasm was higher than that in stress fibers, the difference was not statistically significant between the dimer GFPs and either the monomer or trimer GFPs. Meanwhile, there were significant differences between GFPs in the open cytoplasm and below the nucleus for dimer and trimer GFPs. When comparing the diffusion rates between the GFPs in the open cytoplasm and below the nucleus for dimers and trimers, the GFPs below the nucleus always diffused slower.

**Figure 7:**
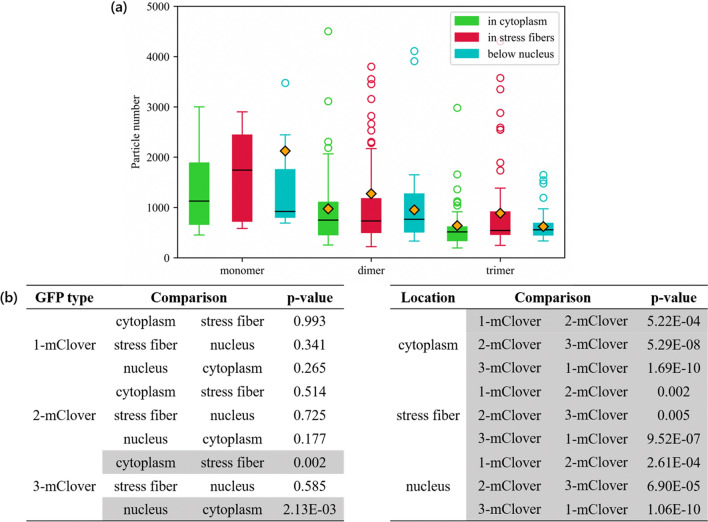
Macromolecular crowding effect on the number of diffusing proteins. **a** Comparison of the number of diffusing particles based on location (cytoplasm, stress fiber, and nucleus). Lower and upper box boundaries represent the 25th and 75th percentiles, respectively. The line inside represents the median, while the diamond represents the mean. The lower and upper error lines are the 10th and 90th percentiles, respectively, and the circles plotted outside these percentiles are the outliers. **b** Table of the statistical analysis performed, where the values in gray highlight the comparisons between datasets with a significance level of *p* < 0.01

Lastly, in Fig. 7a, the particle number was assessed across the three different locations in the cell. In the FCS measurement, the number of particles in the observed confocal volume was also computed by using Eqs. (1) and (2). Generally, the dimer and trimer GFPs were significantly lower in particle number compared to the monomer GFP. Moreover, statistical analysis was also performed, and the resulting *p*-values obtained are listed in Fig. 7b. Here, the gray rows highlight the *p*-values lower than 0.01. Statistical analysis showed that there were significant differences in the data when comparing the number of particles among GFP types. However, in terms of the comparison among GFP in the three subcellular regions, there were no significant differences between the GFP particle numbers.

## Discussion

High macromolecular crowding can greatly impede the way biomolecules diffuse and the resulting extent of their molecular reactions in certain subcellular areas [46–49]. Motivated by previous studies, it is expected that proteins that diffuse in or through stress fibers would be lowered in diffusion as there may be physical obstacles or a nonspecific steric repulsion exerted by the stress fibers onto these surrounding diffusing proteins. Furthermore, we expect that the nucleus could work as an additional obstacle to limit the molecular movement or even physically compress the stress fibers below the nucleus, resulting in an even lower diffusion rate in the cell bottom below the nucleus.

First, FCS was performed to extract the GFP intensity information depicted in Fig. 4, which allows us to evaluate the trends in the diffusion rate among the three subcellular areas. From these average GFP intensity values measured over 20 seconds, there is no significant difference when comparing across the three cell locations. In other words, the fluorescence intensity emitted by the GFPs was more or less around the same values regardless of the cell location. However, although the intensity values are similar, they result in diffusion values that are significantly different from each other when comparing among cellular locations. The diffusion rate was first quantified using the autocorrelation function in Fig. 5 to find that GFP diffusion was the fastest in the cytoplasm among the three subcellular regions investigated. Specifically, the autocorrelation curve declined faster compared to the other two areas. After taking the actual diffusion coefficients in Fig. 6a, the average diffusion coefficients of the GFPs in the cytoplasm was 12.8 μm^2^/s for monomers, 14.7 μm^2^/s for dimers, and 14.6 μm^2^/s for trimers. Following the diffusion in the cytoplasm was the diffusion in the stress fibers, which was lower than that in the cytoplasm by 0.8 to 1.5 μm^2^/s. The slowest diffusion was observed below the nucleus, where the average diffusion was slower than the diffusion in the stress fibers by 0.1 to 1.6 μm^2^/s. However, referring to Fig. 6b, the diffusion coefficients were only significantly different between the GFPs in the nucleus and the cytoplasm for the dimer and trimer GFPs. These results suggest that at the very least, although this was only apparent for dimers and trimers, GFPs below the nucleus diffuse slower compared to the GFPs in the open cytoplasm.

Further, it was assumed that more particles could be observed in crowded stress fibers compared to those in the free cytoplasm comprised of a loose actin meshwork, given that the former would be relatively unable to move freely. However, based on the particle number comparison in Fig. 7, there was no correlation between the number of particles to the different observed locations. We initially hypothesized that, because the presence of intracellular actin-based structures may impede diffusion, the GFPs in stress fibers comprised of high levels of actin filaments may have a lower rate of diffusion. In addition to this reduction in diffusion, the intracellular macromolecular crowding in the packed stress fibers may prevent other surrounding molecules from entering and diffusing across. Thus, the narrow space inside of macromolecules like stress fibers may lower both the diffusion as well as the number of diffusing proteins present, potentially resulting in a lack of net impact in the observable number of the GFPs.

Regarding the diffusion properties, it is explained in other studies that mechanical obstructions, specifically due to the filamentous structures of the cytoskeleton, are what constitute the primary barrier for protein mobility. A previous study explains by using fluorescence recovery after photobleaching (FRAP) method, an alternative to studying protein diffusion using FCS, that the actin cytoskeleton does inhibit protein diffusion for Dictyostelium cells [50]. In the present study, on the other hand, there was only a consistent significant decrease in diffusion observed in the stress fibers below the nucleus. Below the nucleus, it was reported that the space free for movement is limited by the spatial confinement caused by the nucleus, potentially intensifying crowding effects of actin-based structures on diffusing GFP molecules [34]. Although it is not fully certain that macromolecular crowding of stress fibers alone has an influence on the diffusive properties of GFPs, these results are in agreement with the hypothesis as diffusion is delayed in the densely packed stress fibers below the nucleus [34, 35, 33].

The extent of the protein diffusion or intracellular material transport may be associated with the function of stress fibers. Stress fibers are supposed to adaptively disassemble in response to a quick reduction in cellular tension, but because stress fibers are thick in width with highly-packed actin filaments, the interior of the bundled structures may have limitations in interacting with the surrounding diffusing molecules such as cofilin to be promptly unbundled or severed [51]. We previously showed that the individual actin filaments within the bundled stress fibers get separated from each other upon a sudden release of tension to consequently yield a “birdcage” effect where the strands are peeled off [22]. Given the delayed diffusion of proteins inside stress fibers found in the present study, the birdcage effect may be necessary to expose the inside to the surrounding environment to compensate for the limitation in diffusion because a prompt disassembly of stress fibers is key for cellular adaptation to changes in the surrounding environment [52, 53]. Thus, our findings are consistent with the adaptive natures of stress fibers with the highly packed structures.

In addition to studying how protein diffusion rates vary depending on the location of diffusion in the cell, we also focused on the effects of GFP size and mass on protein diffusion as some studies suggest that protein diffusion is dependent on size, and in most cases, heavier molecules diffuse slower [5]. However, other researches on the size dependence of GFP tags on diffusion indicate that although there was a trend of lowering diffusion, the addition of one to four GFP molecules does not result in a significant decrease in diffusion compared to predicted values [54]. As a result, in the second part of the study, we compared the differences in the diffusion rates among three different GFP types (monomer, dimer, and trimer) with various sizes and molecular masses.

Looking at the average intensity values in Fig. 4, there was a significant difference when comparing the monomer intensities to either the dimer or trimer intensities for all cell locations. The monomer GFPs were the lowest in intensity for all cell locations, while the dimer and trimer GFP intensities were comparable. Just from this information alone, the monomer GFPs were more likely to be dispersed compared to dimers or trimers because of their smaller size. However, this alone is not sufficient basis due to the inconsistency of the median and the outliers. Based on the particle number data in Fig. 7, the highest number of molecules in a certain area was the monomer GFP, followed by the dimer GFP and the trimer GFP. This is in line with expectations as larger particles have a higher occupied volume. However, it is interesting to note that the monomer does not double in particle number compared to the dimer, and does not triple in particle number compared to the trimer. Combining these results with those from the intensity profiles, although the dimer and the trimer were comparable in intensity, the dimers were slightly higher in particle number.

Comparing the GFP diffusion across the three different GFP types as in Fig. 6b, there was no clear trend among the monomer, dimer, or trimer GFP diffusion as the majority of the differences among these data were not statistically significant. This means that the diffusion values were more or less the same for the monomer, dimer, and trimer GFP. Although there are studies that conclude that protein diffusion is dependent on size, some researches suggest that there is not much difference in diffusion in this scale. One study compared the diffusion coefficients of engineered GFP multimers, ranging from 2 to 6 covalently linked GFP molecules, in the cytoplasm [54]. Their data suggests that proteins tagged with up to approximately 111 kDa of GFP do not encounter significant diffusion barriers due to macromolecular crowding in the cytoplasm. The mean diffusion coefficients only went below the expectations of the Stokes-Einstein equation when there were five or more linked GFP molecules. Because of this, comparing not only one to three linked GFP molecules but also multimers consisting of four or more linked GFP molecules will be the subject of future investigation.

These results allow for a better understanding of the subcellular protein diffusion and, in turn, mechanical properties of individual adherent cells given that intracellular force measurement using optical tweezers is typically affected by the local structure of the cytoplasm [55]. Researches on macromolecular crowding, size dependence, and other factors that affect protein diffusion have also an impact in different fields including the field of regenerative medicine, with applications in drug delivery. For example, tissue engineering requires the delivery of proteins which relies on the ability to design delivery vehicles that can provide controlled protein release and retention [56]. As these delivery systems are essentially diffusion-controlled, the ability to experimentally determine diffusion coefficients could provide more accurate information than theoretical diffusion coefficients often determined under ideal assumptions.

However, the generality of our findings on the intensity, diffusion, and particle number is subject to several limitations. The proteins used in this study were assumed to be spherical in shape for simplicity. Although this justifies the use of the autocorrelation function for obtaining the diffusion coefficients, this does not consider the effect of different shapes on diffusion. The three-dimensional structure and thus the shape of a protein have an influence on its mobility [57]. The discrepancies in the data could be attributed not to just the size and mass of the GFP, but also to the shape of the GFP, which was not quantitatively evaluated in the scope of this research. In fact, GFP is represented as a compact barrel-shape containing 238 amino acids with 11 beta-strands and an alpha-helix through its center. GFP also has a molecular mass of 27 kDa and has dimensions of 4.2 nm in length and 2.4 nm in diameter, meaning that in theory GFP would obey a more ellipsoidal shape. It must be noted that when such a realistic shape is pursued, some justifying assumptions must also be considered; for example, the linkers between the GFP molecules for the dimer and trimer GFPs should be rigid to prevent any deviations from the ellipsoidal shape. The analysis using such elaborate models will be the subject of future investigation.

## Conclusion

Analyzing GFP diffusion behavior using FCS can lead to a better understanding of the diffusion mechanics of proteins within cells. Here, the effect of macromolecular crowding was observed in dimer and trimer GFPs, and the presence of more actin filaments caused a decrease in the diffusion. The rate of diffusion was dependent on the location of the proteins within the cell, showing that GFP diffuses slowest when located below the nucleus due to the specific macromolecular crowding of the actin meshwork, and the quickest diffusion occurs when GFPs are located in the cytoplasm far from actin stress fibers. However, comparing the GFP diffusion across the three different GFP types showed no clear trend among the monomer, dimer, and trimer GFP diffusion. Nevertheless, it was clear from statistical analysis that macromolecular crowding impacts the extent of protein diffusion in the cell, giving new insights into the cell biological process. Particularly, the finding of the difference in the extent of intracellular material transport within stress fibers seems to be associated with their function. For example, the enlargement of stress fibers and subsequent aggregation of the cell interior in the senescent state could restrict protein diffusion, thereby potentially leading to the reduced turnover and energy consumption as commonly observed in senescent cells.

## Data Availability

The original contributions presented in the study are included in the article material, and further inquiries can be directed to the corresponding author.
